# Supplementary material for: Large bipolaron density at organic semiconductor/electrode interfaces
Source: Nat Commun. 2017 Dec 21;8:2252. doi: 10.1038/s41467-017-02459-3 (PMC5740113; doi:10.1038/s41467-017-02459-3)
Supplement: Supplementary file 1 — Supplementary Information [file 41467_2017_2459_MOESM1_ESM.pdf]

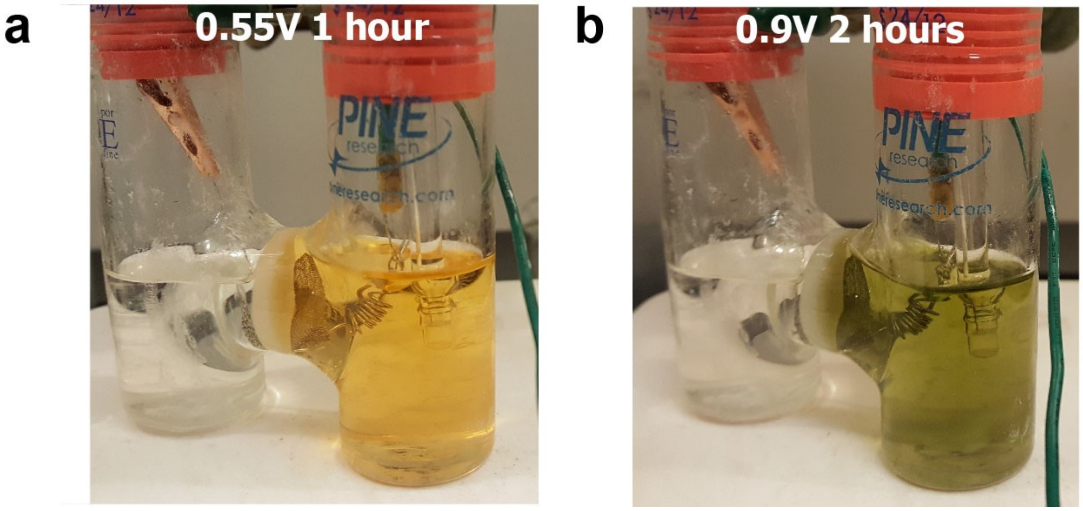


**Supplementary Figure 1 | Spectroelectrochemistry of TPD. a,** Picture of the H-cell after oxidation at 0.55 V relative to a $Ag/Ag^{+}$ reference electrode for one hour, which turns the initially clear TPD solution orange due to absorption by the TPD cation. The $Ag/Ag^{+}$ reference electrode consisted of 0.01 M $AgNO_3$ and 0.1 M tetrabutylammonium tetrafluoroborate electrolyte in acetonitrile. **b,** Oxidizing the same solution at 0.9 V for two hours subsequently changes the color to dark green due to absorption from the TPD dication. Once formed, the orange-colored TPD cation solution is stable for several hours; however, the dication solution reverts back to an orange color in less than 30 minutes.

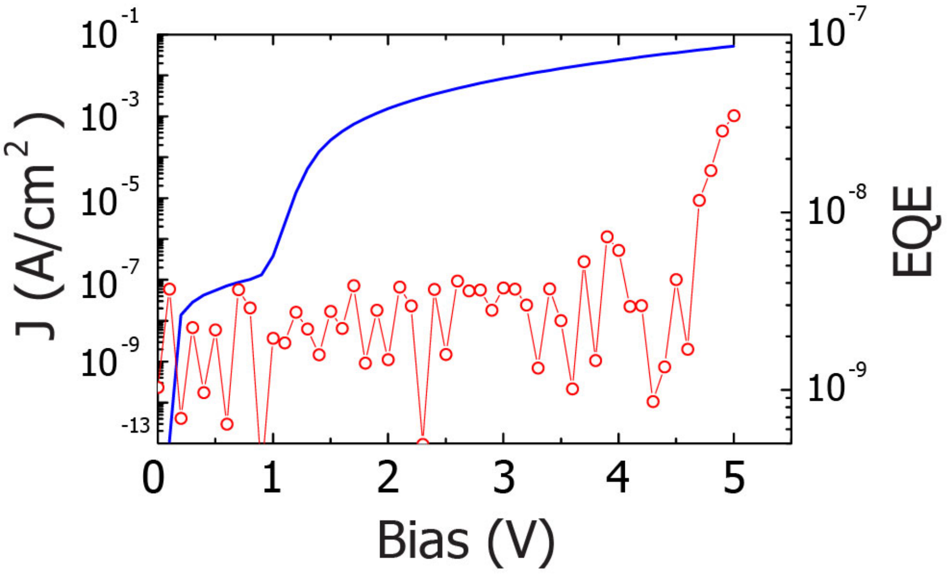


**Supplementary Figure 2 | Unipolar device operation.** Current density and external quantum efficiency (EQE) versus voltage for a typical plasma-treated ITO (100 nm)/TPD (250 nm)/Ag (100 nm) device. Light emission is below the noise floor of the detector over most of the bias range and becomes just barely detectable above ~4.6 V. It is straightforward to estimate the degree of unipolar operation via the EQE according to the usual relationship for organic light emitting diodes,[1] $\mathrm{EQE} = \phi_{\mathrm{B}}\chi_{\mathrm{S}}\eta_{\mathrm{PL}}\eta_{\mathrm{oc}}$. In this expression, $\phi_{\mathrm{B}} = J_{\mathrm{rec}}/J_{\mathrm{tot}}$ is the charge balance factor defined as the ratio of the recombination ($J_{\mathrm{rec}}$) to the total current density ($J_{\mathrm{tot}}$) flowing in the device,[2] $\chi_{\mathrm{S}} = 0.25$ is the fraction of spin-singlet excitons formed upon geminate recombination, $\eta_{\mathrm{PL}} \approx 0.35$ is the TPD photoluminescence quantum yield,[3] and $\eta_{\mathrm{oc}} \approx 0.2$ is the optical outcoupling efficiency of the device. Since any electrons injected at the cathode are likely to recombine with majority holes before reaching the anode, the electron current density $J_{\mathrm{n}} \approx J_{\mathrm{rec}}$ and therefore $J_{\mathrm{n}} < (10^{-5})J_{\mathrm{tot}}$ based on the EQE upper limit of ~$10^{-7}$ in the plot.

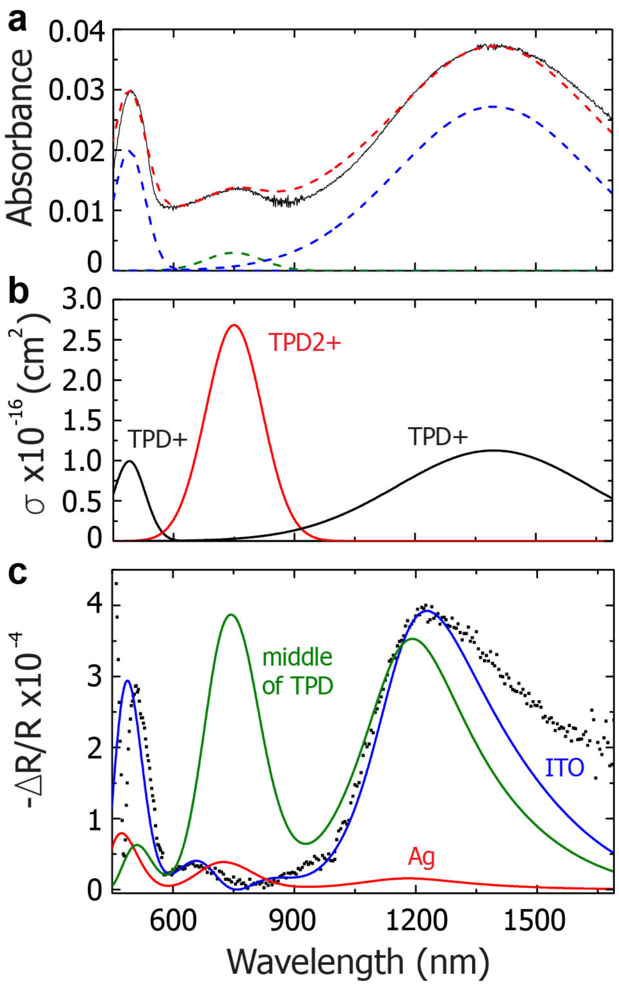


**Supplementary Figure 3 | Optical modeling of charge modulation spectra.** The experimental charge modulation spectra are modeled using the transfer matrix method and the complex refractive index dispersions determined for each layer in the device via spectroscopic ellipsometry. The polaron and bipolaron lineshapes (blue and green dashed lines, respectively) used in the model are determined from a Gaussian multipeak fit of the absorbance spectrum from a 10 wt% $MoO_3$:TPD film as shown in **a**. Their magnitudes are scaled according to the molar absorption coefficients measured from solution to yield the absorption cross-section $[\sigma(\lambda)]$ spectra plotted in **b**. The polaron and bipolaron densities enter the transfer matrix model as an artificial imaginary component of the

complex refractive index, $n + ik$, of TPD (which is otherwise lossless in the visible and near-infrared spectral region) according to $k = (\lambda/4\pi)(\sigma^{+}P^{+} + \sigma^{2+}P^{2+})$; the associated change in the real index of TPD due to the Kramers-Kronig relation is neglected since the magnitude of $k$ is very small. The polaron and bipolaron densities are subsequently determined by assuming a particular location/distribution for each and then varying $P^{+}$ and $P^{2+}$ to generate a reflectivity difference spectrum, $\Delta R/R$, (i.e. the change in reflectivity relative to that calculated with $P^{+} = P^{2+} = 0$) that matches the experimental data. Panel **c** shows an example of how the assumed position of the polaron/bipolaron density leads to interference-related changes in the $\Delta R/R$ spectrum for a plasma-treated ITO (100 nm)/TPD (200 nm)/Ag (100 nm) device for fixed values of $P^{+}$ and $P^{2+}$. The blue spectrum is calculated assuming both species are located in a 1 nm thick region adjacent to the ITO anode whereas the green and red spectra are calculated assuming this 1 nm thick region to be located in the middle of the TPD layer and at the interface adjacent to the Ag cathode, respectively. A uniform distribution throughout the TPD layer is roughly an average of these three spectra. Based on the amplitude of the $\lambda\sim 500$ nm relative to the $\lambda\sim 1400$ nm polaron peak in comparison with the data, it is clear that the polarons must be located close to the anode. Similarly, the position of the bipolaron peak at $\lambda\sim 650$ nm indicates that it too must be located close to the anode in order to match with the data. Taken together with the fact that bipolarons must necessarily exist together with the highest polaron density since the former is a bimolecular product of the latter, it is clear that the bipolarons must be located near the ITO interface.

a

b

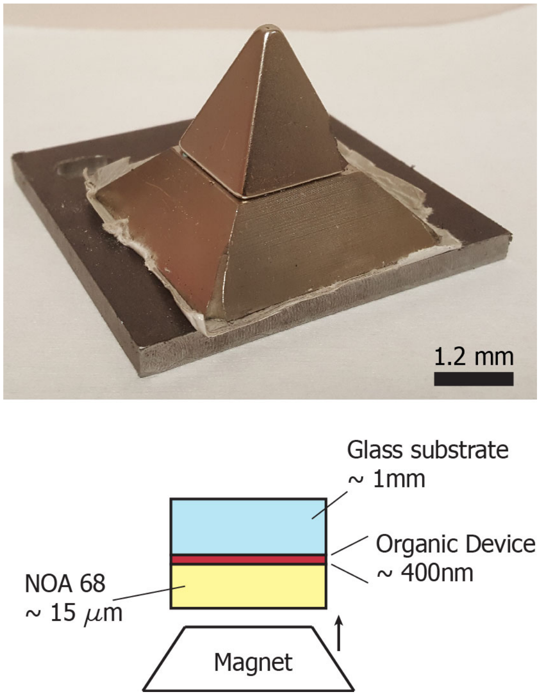


**Supplementary Figure 4 | Experimental details of magnetic field application. a,** Photograph of the permanent N50 neodymium pyramid magnet (PYR1000N and PYR2050N purchased from www.super-magnetman.com and stacked together) used in our experiments. The concentrated magnetic flux density at the surface of the tip exceeds 0.5 T. **b,** Diagram showing how the magnetic field is 'turned on' and 'turned off' by moving the magnet close to, and away from, the device. The magnet is mounted on a translation stage and is moved into direct contact with the ~15 μm thick Norland optical adhesive (NOA 68) encapsulation layer to ensure a reproducible flux density for each forward/backward cycle.

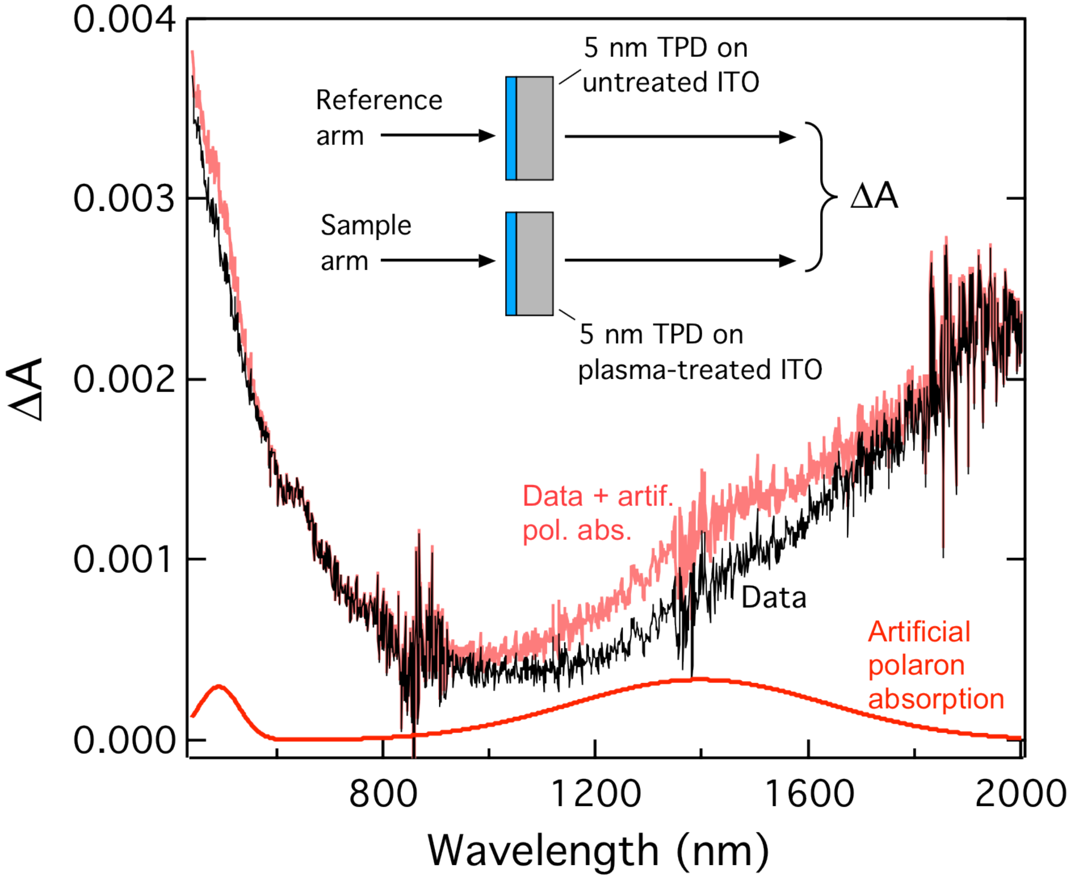


**Supplementary Figure 5 | Bound on equilibrium polaron concentration.** In order to estimate the interfacial polaron density that exists at zero bias in the plasma-treated ITO device, we sought to directly measure the polaron absorbance of a 5 nm thick film of TPD on plasma-treated ITO, using the same film on untreated ITO as a reference (see inset) for 'zero' since the polaron density is much lower in the latter case according to Fig. 3a. The result of this experiment is the black line above, which mainly reflects the change in free carrier concentration associated with oxygen plasma treatment and, notably, does not show clear evidence of the TPD polaron absorption spectrum (red line, reproduced from Fig. S3a). These data consequently provide a bound on the maximum polaron concentration that could exist without leading to a discernable polaron absorption signature within the noise of this particular measurement. The light pink line, which

represents the sum of the black experimental data and the polaron absorbance shown in red, provides a rough indication of the polaron absorbance ($\Delta A \sim 0.0003$ at the $\lambda = 1400$ nm infrared polaron peak) that would be perceptible in this experiment. Based on the associated absorption cross-section, $\sigma$, at the same wavelength in Fig. S3b, the equilibrium interfacial polaron concentration must be less than $P^{+}_{\max} \sim \ln(10)\Delta A/\sigma = 6\text{x}10^{12}$ cm$^{-2}$. We note that the equilibrium polaron concentration at zero bias could have been determined more directly from the data in Fig. 3a by continuing in reverse bias to the point at which $\Delta P^{+}$ stopped decreasing and became flat (which would correspond to completely depleting the interfacial charge). Unfortunately, it was not possible to reach this point because $\Delta P^{+}$ continued its linear decrease in reverse bias up until the point of dielectric breakdown, typically at about -20 V. Based on the slope of the decrease for, e.g. the plasma-treated device (where $\Delta P^{+}/\Delta V \sim 10^{10}$ cm$^{-2}$V$^{-1}$ in Fig. 3a), this is not unexpected since it would take a bias of -600 V to fully deplete a concentration such as $P^{+}_{\max}$ estimated above. By contrast, the differential bipolaron concentration, $\Delta P^{2+}$, in Fig. 2b does exhibit a flat slope below ~3 V and thus can straightforwardly be interpreted as the total concentration (i.e. $\Delta P^{2+} = P^{2+}$).

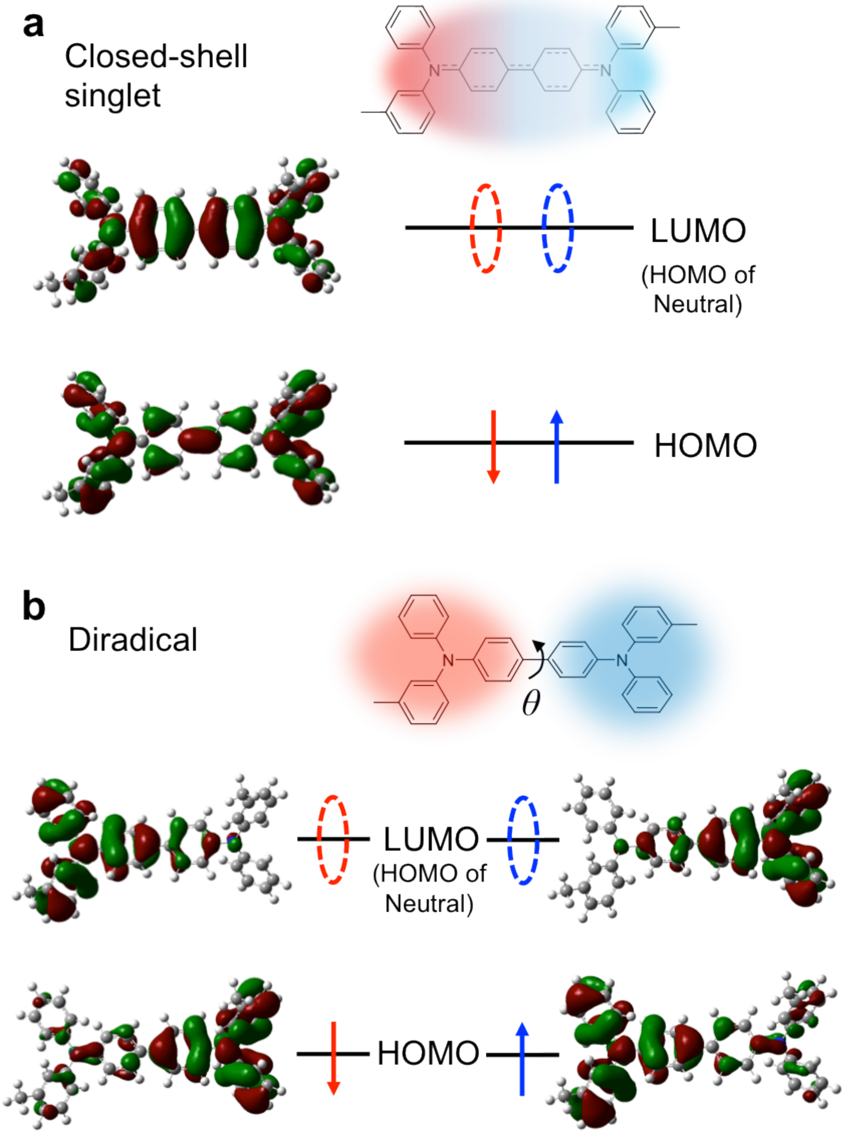


**Supplementary Figure 6 | DFT predictions for the bipolaron.** Geometries for the TPD neutral molecule, radical cation, and dication are calculated using density functional theory (DFT). These calculations indicate two potential states for the dication: a closed-shell singlet state where both holes (dashed ovals in the energy level diagram) delocalize over the molecule as shown in panel **a**, and a diradical state, where a $\theta \sim 27°$ dihedral twist angle breaks the conjugation and allows the holes to localize on opposite aryl-amine fragments as indicated by the red and blue shading in **b**. The Hubbard energy of the

diradical state is predicted to be roughly 0.1 eV lower than that of the closed-shell singlet due to the node in the wavefunction, which reduces Coulombic repulsion between the two holes. Given that the reorganization energies of each state calculated relative to the cation are similar (0.06 eV difference), it therefore seems likely that the bipolarons observed in our experiments exist in the diradical state.

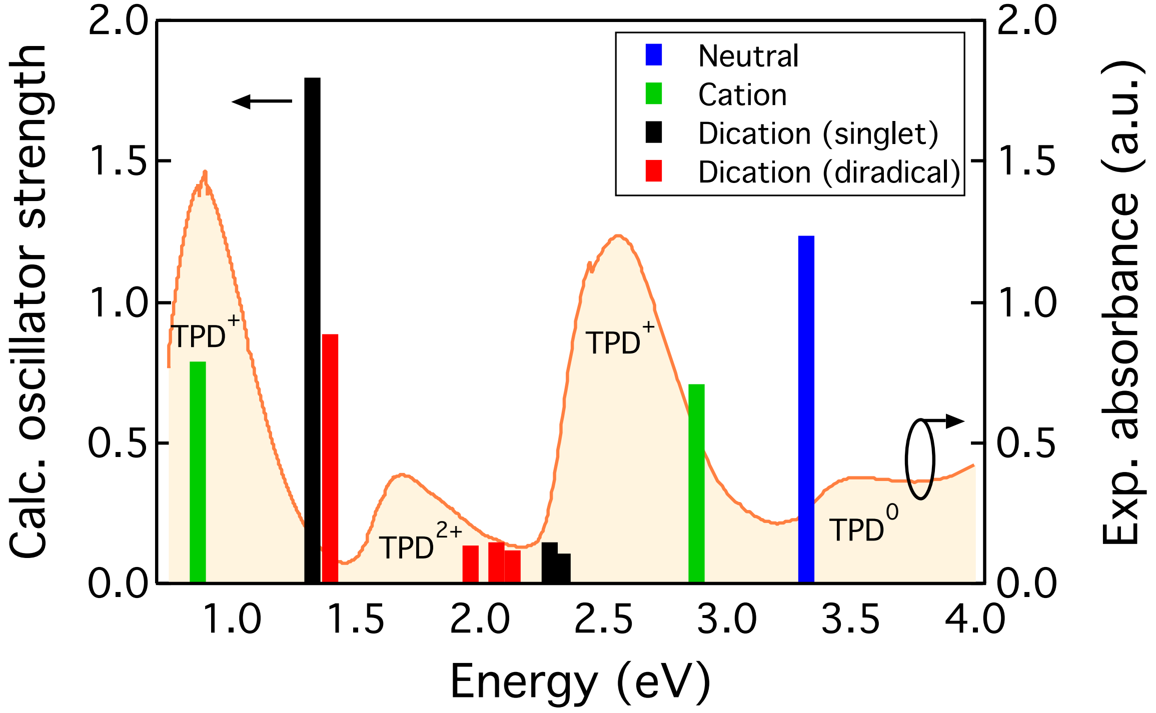


**Supplementary Figure 7 | TDDFT calculated optical transitions.** Comparison of the optical transitions predicted via time-dependent density functional theory (TDDFT) for the TPD neutral molecule and its various charged species. The experimental absorption spectrum obtained from solution at -0.9 V is reproduced from Fig. 1a on the right-hand axis for comparison. We observe reasonable overall agreement within a few tenths of an eV between the predicted and observed transitions. At this level of accuracy, however, the similarity between the predicted diradical and closed-shell singlet transitions does not allow them to be distinguished spectroscopically.

**Supplementary References**


1 Adachi, C., Baldo, M. A., Thompson, M. E. & Forrest, S. R. Nearly 100% internal phosphorescence efficiency in an organic light-emitting device. *J. Appl. Phys.* **90**, 5048-5051, (2001).

2 Ruhstaller, B., Carter, S. A., Barth, S., Riel, H., Riess, W. & Scott, J. C. Transient and steady-state behavior of space charges in multilayer organic light-emitting diodes. *J. Appl. Phys.* **89**, 4575-4586, (2001).

3 Mattoussi, H., Murata, H., Merritt, C. D., Iizumi, Y., Kido, J. & Kafafi, Z. H. Photoluminescence quantum yield of pure and molecularly doped organic solid films. *J. Appl. Phys.* **86**, 2642-2650, (1999).
